# Supplementary material for: Increased hydraulic risk in assemblages of woody plant species predicts spatial patterns of drought-induced mortality
Source: Nat Ecol Evol. 2023 Aug 28;7(10):1620–32. doi: 10.1038/s41559-023-02180-z (PMC10555820; doi:10.1038/s41559-023-02180-z)
Supplement: Supplementary file 1 — Supplementary Figs. 1–10 and Tables 1–5. [file 41559_2023_2180_MOESM1_ESM.pdf]

nature ecology & evolution

Article https://doi.org/10.1038/s41559-023-02180-z

Increased hydraulic risk in assemblages of woody plant species predicts spatial patterns of drought-induced mortality

In the format provided by the authors and unedited

Supplementary material

Contents

Supplementary Figure 1. Hydraulic traits standard deviation. 2

Supplementary Figure 2. P_{min} and/or $P_{50/88}$ phylogenetic distribution. 4

Supplementary Figure 3. Geographical coverage of species distributions and mortality data... 6

Supplementary Figure 4. Geographic distribution of P_{min}. 7

Supplementary Figure 5. Geographic distribution of $P_{50/88}$ and $HSM_{50/88}$. 7

Supplementary Figure 6. Relationship between drought-induced mortality and functional types. 8

Supplementary Figure 7. Geographic distribution of standard deviations. 9

Supplementary Figure 8. Comparison with Trugman et al. (2020). 10

Supplementary Figure 9. Methods scheme. 11

Supplementary Figure 10. Trait distribution excluding non-woody areas. 11

Supplementary Table 1. Random forest models performance. 12

Supplementary Table 2. Prediction of DIM occurrence. 16

Supplementary Table 3. Relationship of hydraulic risk and mortality including aridity index as a covariable. 17

Supplementary Table 4. Number of mortality events observed per biome. 18

Supplementary Table 5. Trends in the relationship between species assemblages hydraulic metrics and DIM occurrence as reported by applying the emmeans R package[2] to generalized linear models results. 19

References 21

Supplementary Figure 1. Hydraulic traits standard deviation.

Hydraulic trait standard deviation calculated from imputation results obtained by iterating the predictive model 100 times. Values are aggregated at the genus level by calculating the mean for genera with more than one species. In red, are taxa with observed mortality. Order names are shown proportional to the number of taxa and marked in the phylogeny by different colours at the tips.

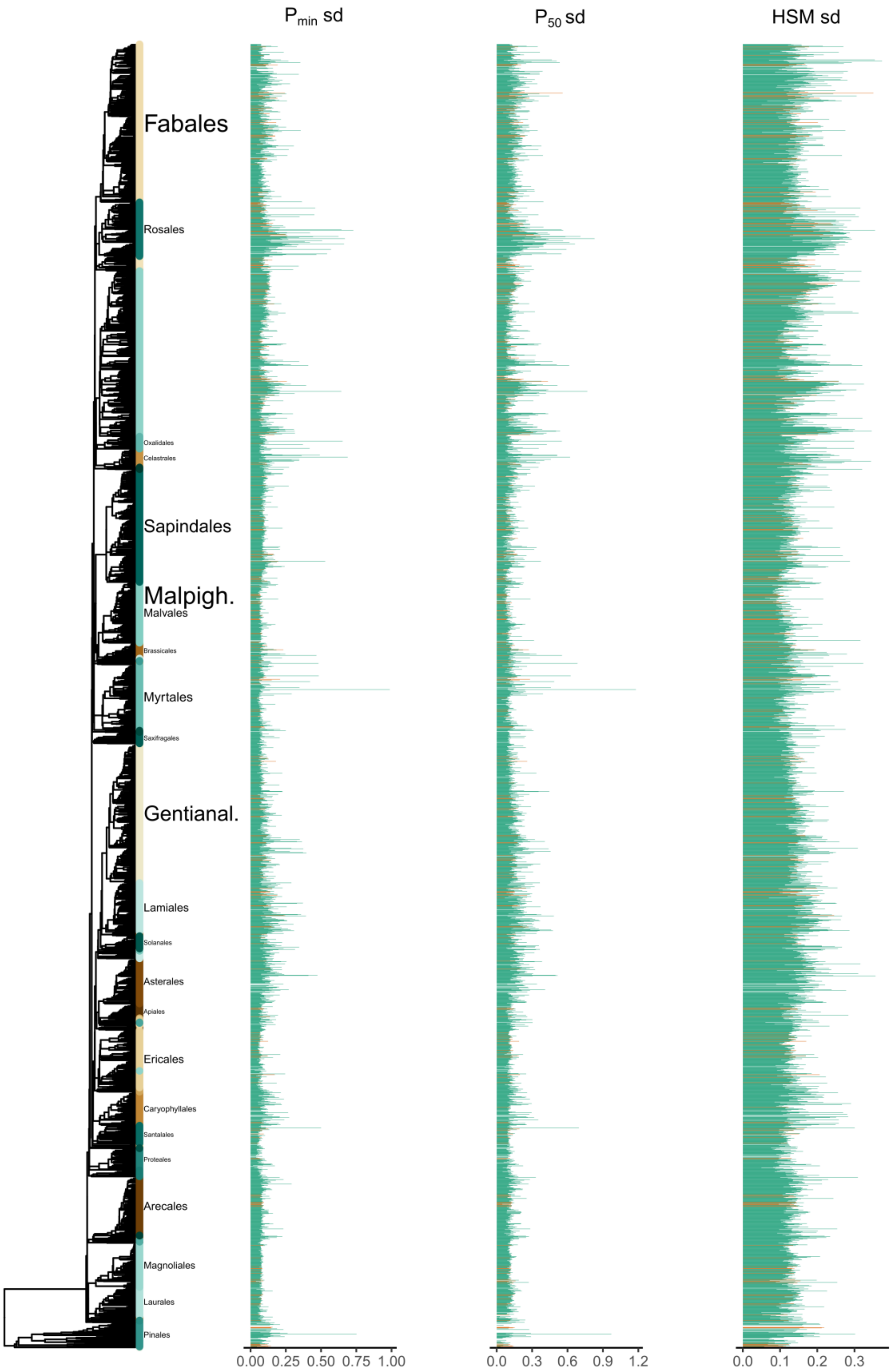

Pmin sd
P50 sd
HSM sd
Fabales
Rosales
Oxalidales
Celastrales
Sapindales
Malpigh.
Malvales
Brassicales
Myrtales
Saxifragales
Gentianal.
Lamiales
Solanales
Asterales
Apiales
Ericales
Caryophyllales
Santalales
Proteales
Arecales
Magnoliales
Laurales
Pinales
0.00 0.25 0.50 0.75 1.00
0.0 0.3 0.6 0.9 1.2
0.0 0.1 0.2 0.3

31

Supplementary Figure 2. P_{min} and/or $P_{50/88}$ phylogenetic distribution.

Phylogenetic distribution of observed and imputed hydraulic traits for species with observed data for P_{min} and/or $P_{50/88}$. Imputed values represent the mean of 100 predicted values per species resulting from 100 iterations of the predictive model. In red, species with observed mortality and in green species without observed mortality. The most important order names are shown with size proportional to the number of species represented. The total number of species with trait data is shown in black and the number in red is the number of those species that have an observed mortality event. The number and percentage of species showing hydraulic safety margin ($HSM_{50/88}$) values below zero, 0.5 and 1 are also shown. Note that number of species with observed mortality differs from the ones showed in figure 1 as the number of species with available P_{50} and P_{88} slightly differed.

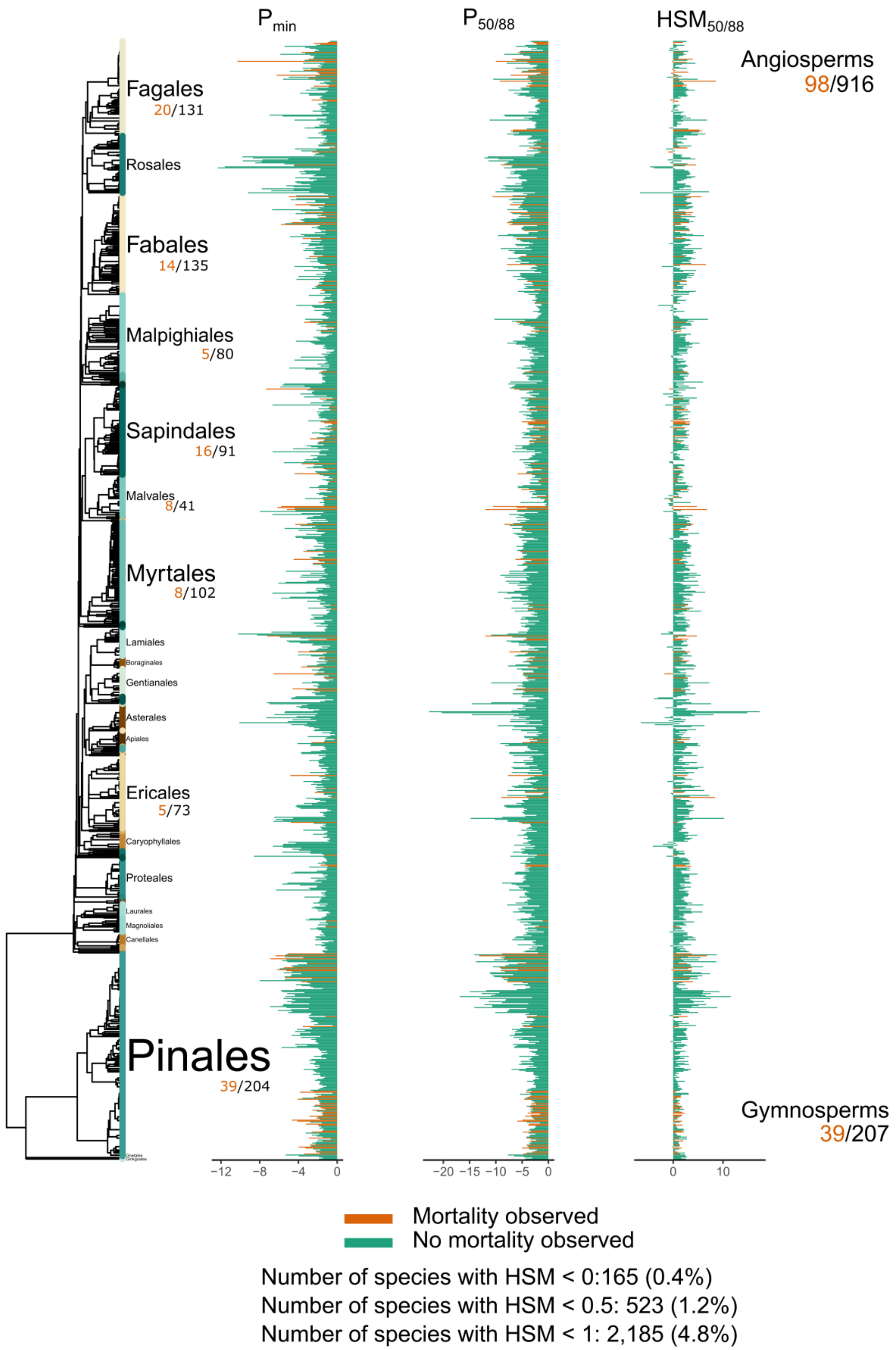
P_{min}
$P_{50/88}$
$HSM_{50/88}$
Angiosperms
98/916
Fagales
20/131
Rosales
Fabales
14/135
Malpighiales
5/80
Sapindales
16/91
Malvales
8/41
Myrtales
8/102
Lamiales
Boraginales
Gentianales
Asterales
Apiales
Ericales
5/73
Caryophyllales
Proteales
Laurales
Magnoliales
Canellales
Pinales
39/204
Gymnosperms
39/207
−12 −8 −4 0
−20 −15 −10 −5 0
0 10
Mortality observed
No mortality observed
Number of species with HSM < 0:165 (0.4%)
Number of species with HSM < 0.5: 523 (1.2%)
Number of species with HSM < 1: 2,185 (4.8%)

Supplementary Figure 3. Geographical coverage of species distributions and mortality data. Geographical coverage of species range distribution data for: a) the number of species for which imputation was implemented and b) for species with observed traits data. C) Mortality points distribution and one of the background sets used in generalized linear models (red and green, respectively) plotted on HSM mean map.

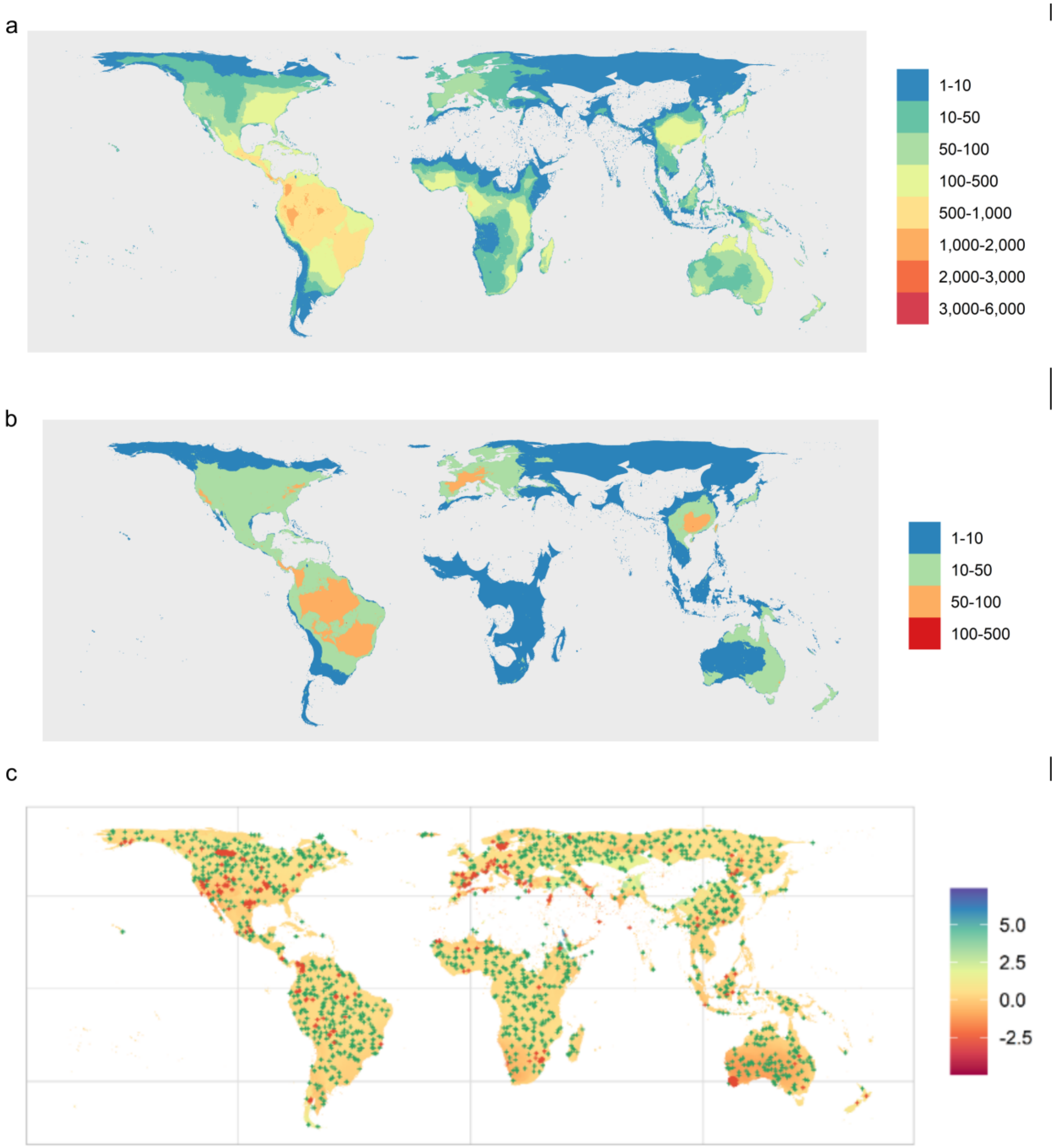

Supplementary Figure 4. Geographic distribution of P_{min}.

Geographic distribution of projected species-assemblage P_{min} means and range and latitudinal patterns ("a" and "b", respectively). Scatterplots show pixel values per latitude as circles and absolute minimum and maximum values by latitude (i.e., accounting for all species-level values present in a pixel) as red and blue triangles in "a". Trend lines for pixel values and absolute maximum and minimum values in "a" are shown following a GAM methodology.

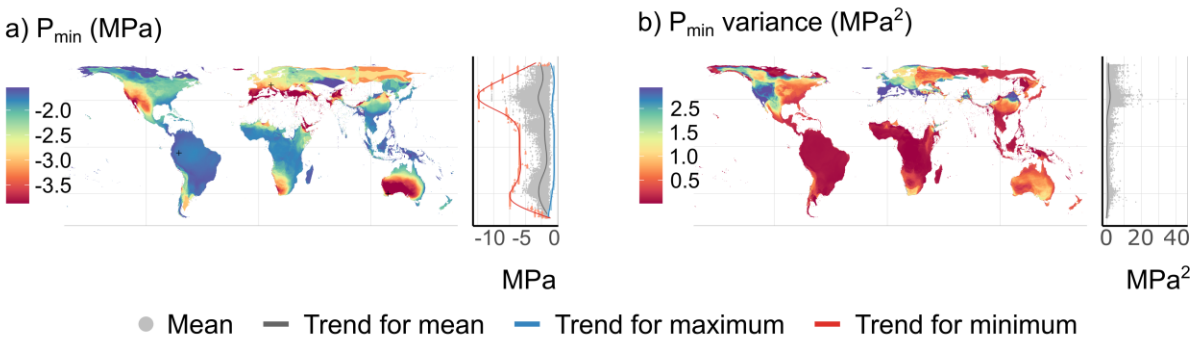

Supplementary Figure 5. Geographic distribution of $P_{50/88}$ and $HSM_{50/88}$.

Geographical distribution of projected species-assemblage hydraulic metrics and corresponding latitudinal patterns. a, b: Mean $P_{50/88}$ and $HSM_{50/88}$, respectively and c: number of species with negative $HSM_{50/88}$ values. The distribution of species-level values from which metrics are calculated for a sample of three representative pixels is shown in histograms in "a" and "b". Scatterplots show the distribution of pixel values by latitude using circles, absolute minimum and maximum values by latitude (i.e., accounting for all species-level values present in a pixel) as red and blue triangles, respectively. Trend lines for pixel values and absolute maximum and minimum values for scatterplots in "a" and "b" are shown following a GAM methodology.

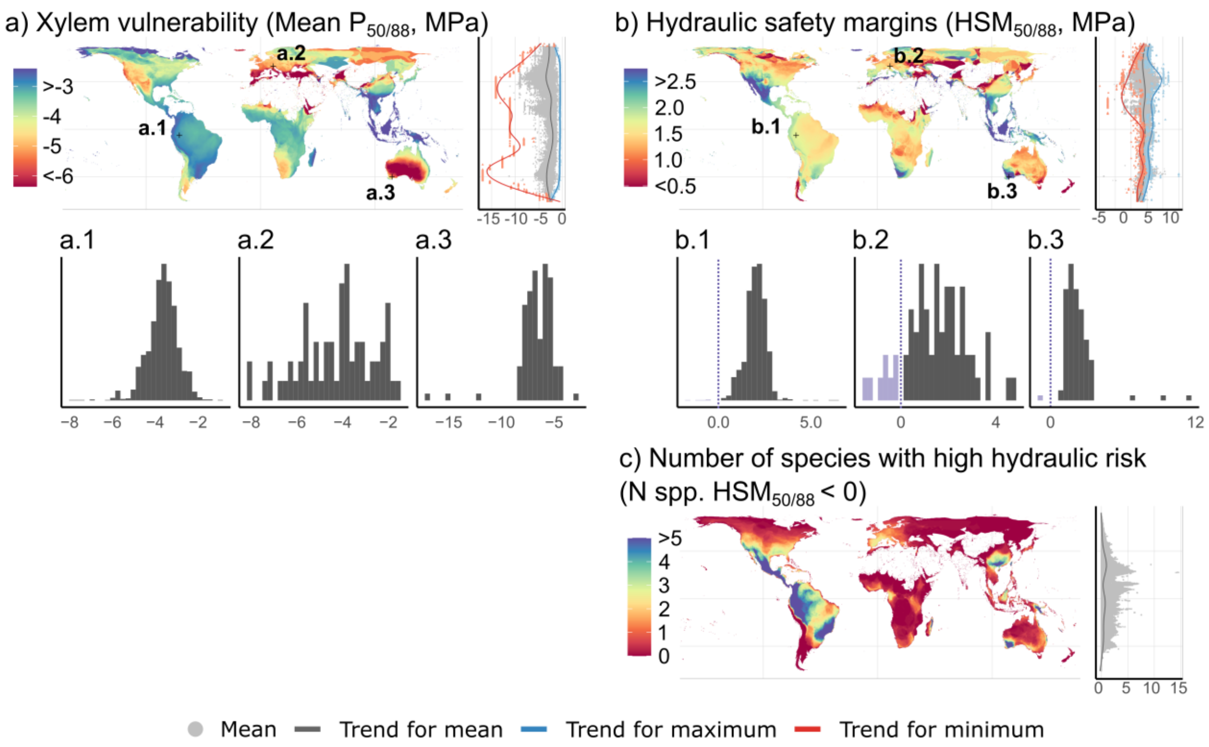

Supplementary Figure 6. Relationship between drought-induced mortality and functional types. Relationship between drought-induced mortality occurrence and species-assemblage hydraulic metrics representing “a”: mean HSM; “b”: minimum HSM; “c”: HSM variability and “d”: number of species with HSM < 0 including their interaction with functional type distribution. Results summarize 100 iterations of each model from which R^{2}, and test AUC mean and standard deviation were calculated. In each iteration, a different set of background points was sampled. Mean response curves and their and the 95% coefficient interval for species-assemblage metrics for each functional type are shown.

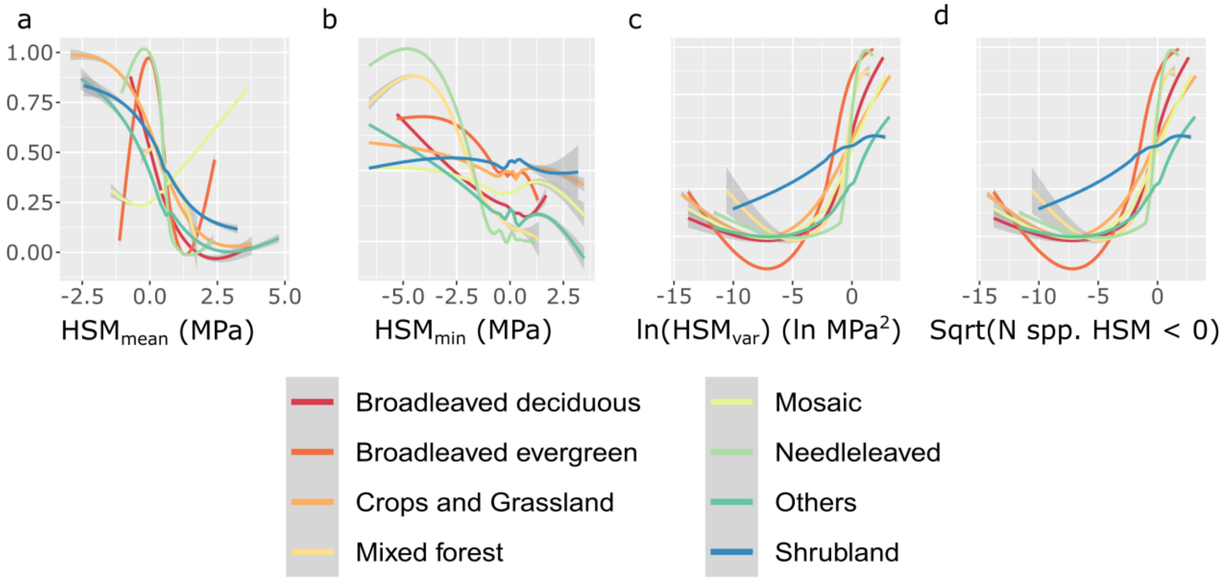

Supplementary Figure 7. Geographic distribution of standard deviations.

Mean of standard deviations resulting from 100 iterations of the predictive models (left column) and from 100 iterations of the predictive models randomly excluding 20% of the species with observed traits in each case (right column) for P_{min} (first row), P_{50} (second row) and HSM (third row).

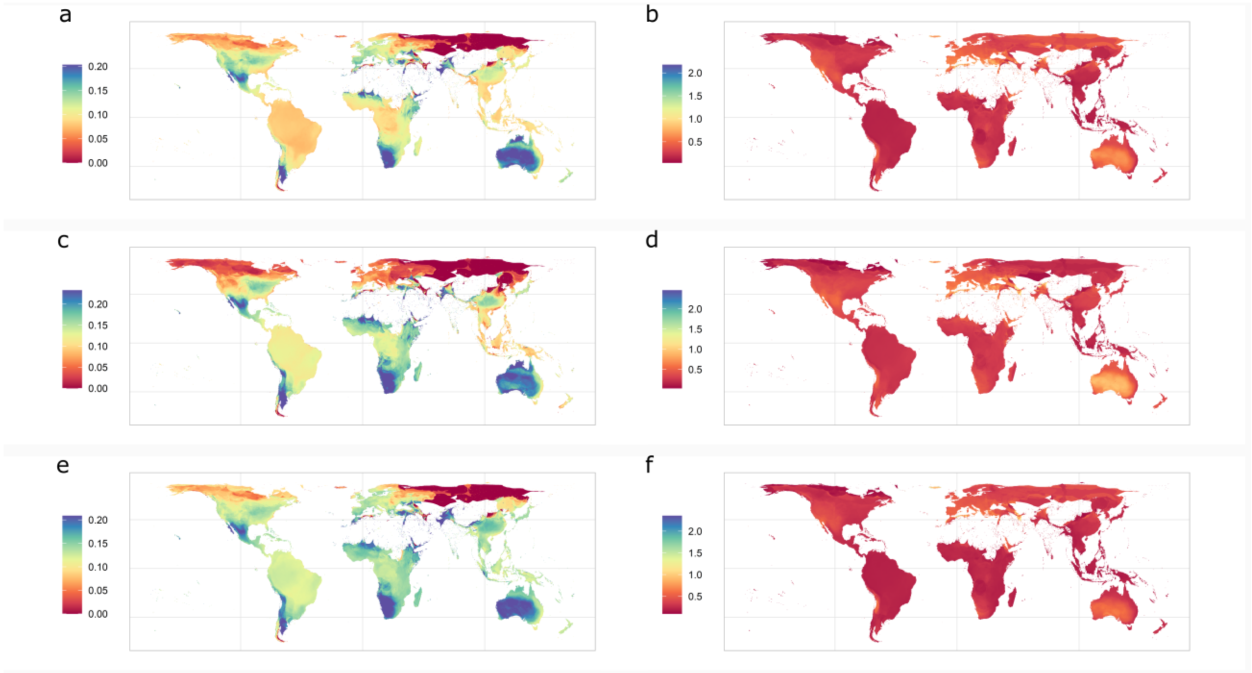

Supplementary Figure 8. Comparison with Trugman et al. (2020).

Comparison between P_{50} and HSM mean and range projections calculated here with previous results including plot-level data[1] for the United States of America. Linear regressions coefficients and R^2 are shown.

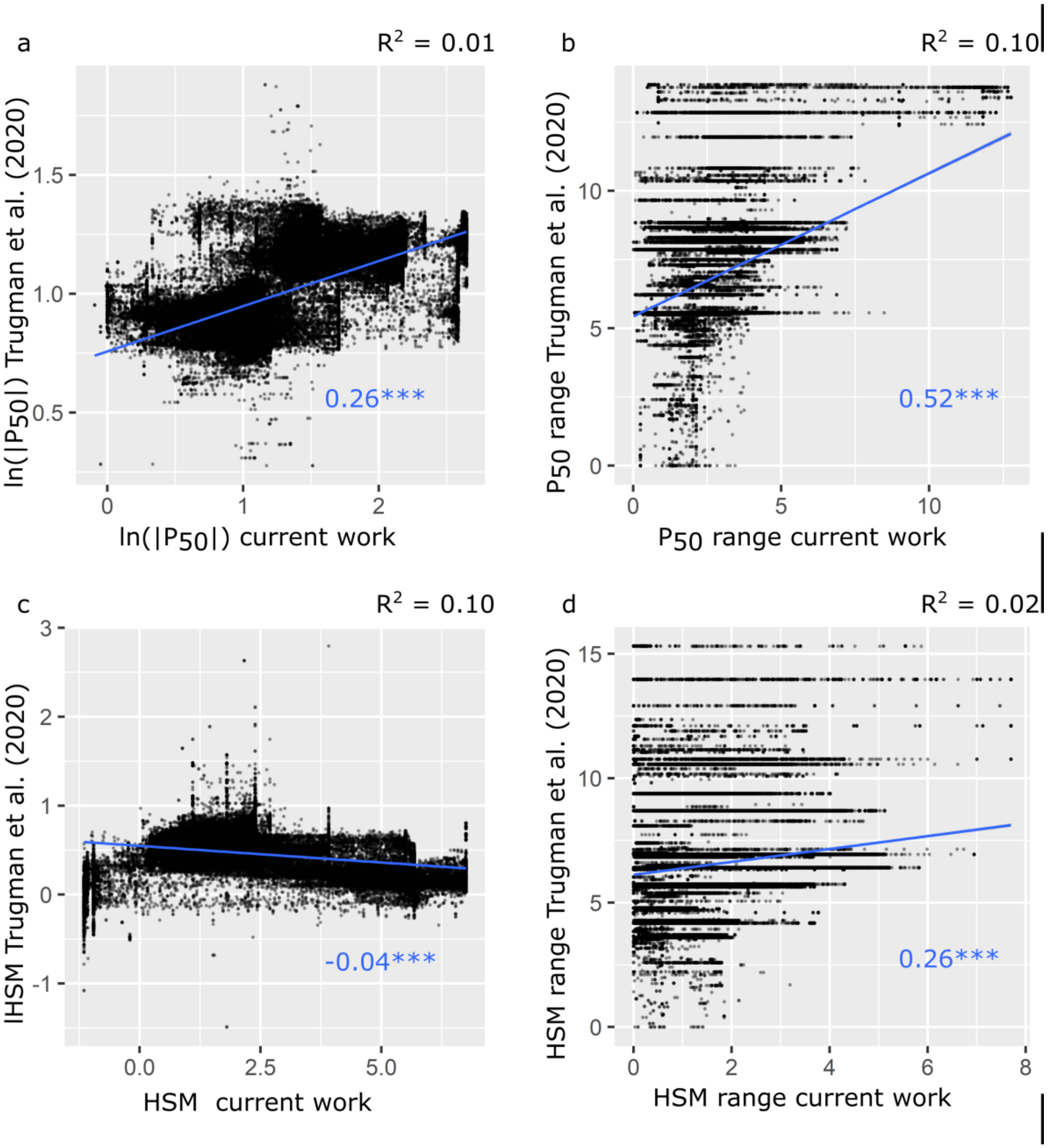

Supplementary Figure 9. Methods scheme.

Scheme of the methodology implemented, and the data used in each case. The data used is shown in black circles. The main results obtained in each step are also referred in red boxes.

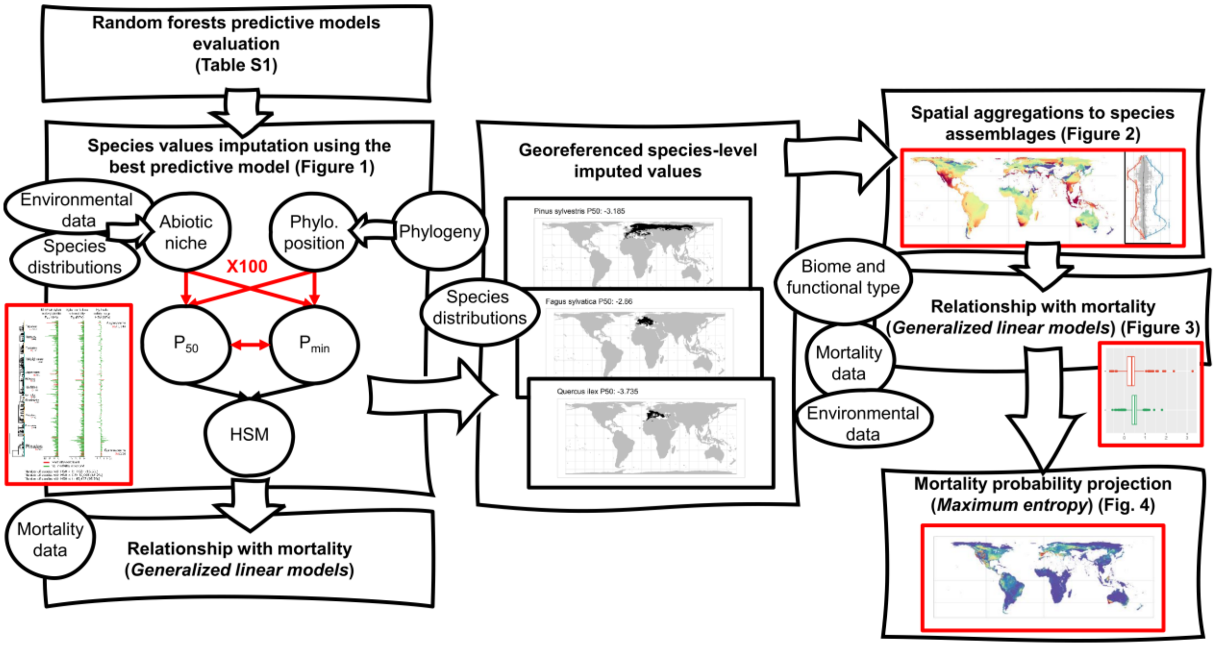

Supplementary Figure 10. Trait distribution excluding non-woody areas.

Global distribution of species-assemblage hydraulic metrics and their latitudinal patterns excluding areas without woody vegetation. “a” shows an example on the hydraulic risk composition for a given species assembly, from which the plotted metrics are calculated. “b”: mean P_{50} and “c”: hydraulic risk represented as negative hydraulic safety margin (-HSM); “d” and “e”: P_{50} and HSM variance; “f”: the number of species with negative HSM values and “g”: maximum hydraulic risk represented as negative minimum HSM. The distribution of species-level values from which metrics are calculated for a sample of three representative pixels are shown in histograms in “b” and “c”. Lateral scatterplots in “b” to “g” show the distribution of pixel values by latitude in grey, and absolute minimum and maximum values by latitude (i.e., accounting for

all species-level values present in a pixel) as red and blue, respectively. Trend lines for pixel values and absolute maximum and minimum values are also shown for scatterplots in “b” and “c”, by means of generalised additive model (GAM).

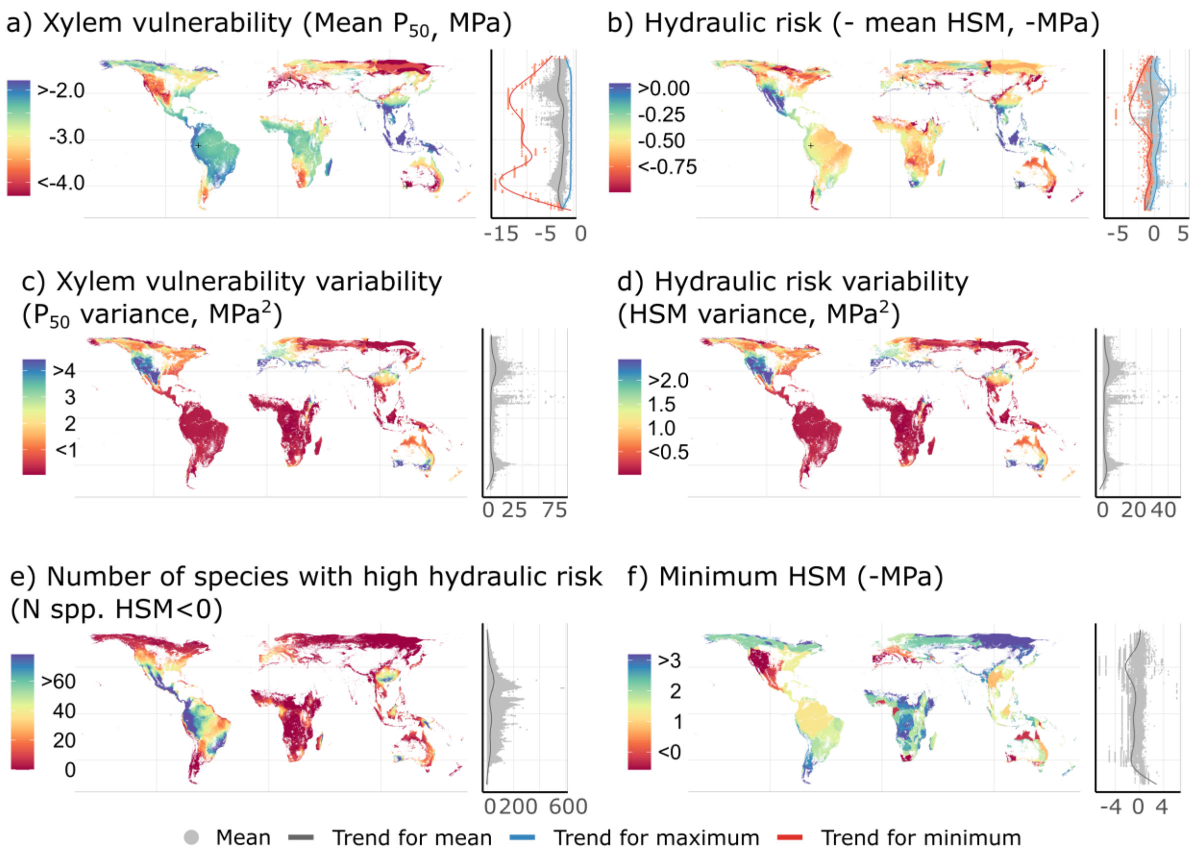

Supplementary Table 1. Random forest models performance.

Random forest models performance. R^2 results for different combinations of predictors and different percentages of data used to train and test models. Model refers to the model tested indicating the variable predicted (bi when both are predicted at the same time), the number of climate (climate_n) and phylogenetic predictors (phylo_n) and whether evolutionary affiliation (angiosperms vs. gymnosperms) was included as an explanatory factor (group), in this order. In bold, the models used for the final prediction.

Model	Variable	N edaphoclimatic PCs	N phylogenetic PCs	Evolutionary affiliation	Hydraulic trait covariance	R2 (10% test)	R2 sd (10% test)	R2 (20% test)	R2 sd (20% test)	R2 (30% test)	R2 sd (30% test)	R2 (50% test)	R2 sd (50% test)	R2 (70% test)	R2 sd (70% test)
P50_climate_3	P_{50}	3	0	No	No	0.11	0.055	0.106	0.038	0.102	0.029	0.095	0.021	0.084	0.017
Pmin_climate_3	P_{min}	3	0	No	No	0.369	0.094	0.35	0.064	0.355	0.052	0.334	0.032	0.318	0.03
bi_climate_3	P_{min}	3	0	No	Yes	0.512	0.123	0.469	0.092	0.441	0.066	0.364	0.051	0.281	0.058
bi_climate_3	P_{50}	3	0	No	Yes	0.304	0.123	0.277	0.081	0.25	0.072	0.183	0.05	0.116	0.038
P50_climate_10	P_{50}	10	0	No	No	0.154	0.071	0.154	0.046	0.143	0.032	0.136	0.025	0.12	0.02
Pmin_climate_10	P_{min}	10	0	No	No	0.415	0.096	0.377	0.06	0.375	0.052	0.364	0.037	0.335	0.03
bi_climate_10	P_{min}	10	0	No	Yes	0.544	0.102	0.499	0.074	0.465	0.061	0.399	0.047	0.337	0.043
bi_climate_10	P_{50}	10	0	No	Yes	0.35	0.122	0.309	0.087	0.263	0.067	0.198	0.047	0.133	0.035
P50_phylo_5	P50	0	5	No	No	0.473	0.088	0.465	0.059	0.453	0.053	0.433	0.031	0.402	0.027
Pmin_phylo_5	P_{min}	0	5	No	No	0.333	0.108	0.3	0.084	0.297	0.061	0.249	0.05	0.191	0.04
bi_phylo_5	P_{min}	0	5	No	Yes	0.457	0.137	0.422	0.099	0.4	0.071	0.311	0.058	0.237	0.049
bi_phylo_5	P_{50}	0	5	No	Yes	0.5	0.108	0.471	0.102	0.45	0.066	0.387	0.053	0.332	0.048
P50_phylo_20	P_{50}	0	20	No	No	0.474	0.097	0.461	0.064	0.451	0.051	0.438	0.034	0.409	0.028
Pmin_phylo_20	P_{min}	0	20	No	No	0.341	0.134	0.313	0.082	0.306	0.058	0.267	0.049	0.206	0.038
bi_phylo_20	P_{min}	0	20	No	Yes	0.438	0.131	0.428	0.095	0.396	0.082	0.338	0.049	0.258	0.047
bi_phylo_20	P_{50}	0	20	No	Yes	0.485	0.159	0.464	0.093	0.454	0.071	0.397	0.052	0.349	0.045
P50_phylo_50	P_{50}	0	50	No	No	0.455	0.106	0.475	0.062	0.462	0.047	0.44	0.038	0.411	0.028
Pmin_phylo_50	P_{min}	0	50	No	No	0.345	0.13	0.311	0.077	0.31	0.067	0.271	0.047	0.206	0.041
bi_phylo_50	P_{min}	0	50	No	Yes	0.439	0.14	0.42	0.096	0.387	0.088	0.336	0.047	0.269	0.041
bi_phylo_50	P_{50}	0	50	No	Yes	0.485	0.123	0.481	0.091	0.44	0.063	0.398	0.047	0.352	0.038
P50_phylo_100	P_{50}	0	100	No	No	0.487	0.088	0.482	0.058	0.461	0.052	0.452	0.03	0.425	0.026

Pmin_phylo_100	P_{min}	0	100	No	No	0.341	0.126	0.318	0.077	0.304	0.069	0.264	0.038	0.206	0.032
bi_phylo_100	P_{min}	0	100	No	Yes	0.44	0.119	0.412	0.105	0.373	0.068	0.326	0.05	0.249	0.042
bi_phylo_100	P_{50}	0	100	No	Yes	0.456	0.132	0.429	0.097	0.429	0.073	0.395	0.044	0.357	0.043
P50_climate_3_phylo_5_group	P_{50}	3	5	Yes	No	0.573	0.101	0.558	0.068	0.556	0.054	0.535	0.029	0.498	0.028
Pmin_climate_3_phylo_5_group	P_{min}	3	5	Yes	No	0.467	0.115	0.468	0.086	0.456	0.06	0.43	0.039	0.401	0.031
bi_climate_3_phylo_5_group	P_{min}	3	5	Yes	Yes	0.588	0.122	0.559	0.082	0.525	0.066	0.475	0.056	0.399	0.049
bi_climate_3_phylo_5_group	P_{50}	3	5	Yes	Yes	0.551	0.123	0.528	0.1	0.515	0.071	0.459	0.055	0.399	0.046
P50_climate_5_phylo_5_group	P_{50}	5	5	Yes	No	0.567	0.101	0.564	0.063	0.563	0.047	0.532	0.035	0.507	0.026
Pmin_climate_5_phylo_5_group	P_{min}	5	5	Yes	No	0.489	0.101	0.491	0.076	0.485	0.052	0.457	0.039	0.425	0.029
**bi_climate_5_phylo_5_group**	$\mathbf{P_{min}}$	**5**	**5**	Yes	**Yes**	0.594	0.118	0.581	0.079	0.551	0.061	0.496	0.051	0.44	0.04
**bi_climate_5_phylo_5_group**	$\mathbf{P_{50}}$	**5**	**5**	Yes	**Yes**	0.547	0.13	0.537	0.091	0.526	0.069	0.476	0.054	0.403	0.045
P50_climate_10_phylo_10_group	P_{50}	10	10	Yes	No	0.579	0.094	0.575	0.057	0.57	0.046	0.538	0.035	0.509	0.021
Pmin_climate_10_phylo_10_group	P_{min}	10	10	Yes	No	0.483	0.108	0.485	0.069	0.464	0.059	0.458	0.036	0.422	0.031
bi_climate_10_phylo_10_group	P_{min}	10	10	Yes	Yes	0.575	0.117	0.564	0.078	0.559	0.062	0.505	0.046	0.434	0.046
bi_climate_10_phylo_10_group	P_{50}	10	10	Yes	Yes	0.558	0.139	0.541	0.087	0.521	0.071	0.489	0.044	0.411	0.038
P50_climate_10_phylo_20_group	P_{50}	10	10	Yes	No	0.569	0.094	0.557	0.066	0.56	0.056	0.542	0.037	0.501	0.028
Pmin_climate_10_phylo_20_group	P_{min}	10	20	Yes	No	0.491	0.101	0.47	0.08	0.47	0.06	0.443	0.043	0.406	0.037
bi_climate_10_phylo_20_group	P_{min}	10	20	Yes	Yes	0.578	0.126	0.578	0.105	0.544	0.067	0.492	0.048	0.435	0.041
bi_climate_10_phylo_20_group	P_{50}	10	20	Yes	Yes	0.551	0.129	0.54	0.087	0.517	0.068	0.458	0.05	0.405	0.04
P50_climate_10_phylo_50_group	P_{50}	10	50	Yes	No	0.572	0.103	0.567	0.067	0.556	0.051	0.533	0.035	0.496	0.026
Pmin_climate_10_phylo_50_group	P_{min}	10	50	Yes	No	0.473	0.13	0.463	0.077	0.459	0.059	0.436	0.045	0.39	0.04
bi_climate_10_phylo_50_group	P_{min}	10	50	Yes	Yes	0.547	0.134	0.528	0.093	0.511	0.077	0.478	0.048	0.411	0.046
bi_climate_10_phylo_50_group	P_{50}	10	50	Yes	Yes	0.534	0.128	0.507	0.098	0.492	0.071	0.452	0.053	0.391	0.04

P50_climate_10_phylo_100_group	P_{50}	10	100	Yes	No	0.557	0.101	0.557	0.062	0.546	0.051	0.524	0.039	0.49	0.025
Pmin_climate_10_phylo_100_group	P_{min}	10	100	Yes	No	0.467	0.111	0.45	0.079	0.449	0.064	0.414	0.046	0.373	0.045
bi_climate_10_phylo_100_group	P_{min}	10	100	Yes	Yes	0.545	0.118	0.512	0.098	0.496	0.07	0.457	0.052	0.389	0.042
bi_climate_10_phylo_100_group	P_{50}	10	100	Yes	Yes	0.497	0.132	0.49	0.097	0.469	0.076	0.441	0.048	0.385	0.039

Supplementary Table 2. Prediction of DIM occurrence.

Pseudo-R^2 test AUC mean and standard deviation and mean model AIC (summary of 100 iterations with different background points) for logistic models with mortality occurrence as a response variable (binary). In the model column, model formula is shown.

Model	Mean R^2	R^2 standard deviation	Mean test AUC	Test AUC standard deviation	AIC
p ~ ln(aridity index)	0.011	0.006	0.590	0.042	1384.780
p ~ annual precipitation	0.016	0.006	0.555	0.036	1381.321
p ~ maximum temperature	0.019	0.008	0.594	0.045	1378.893
p ~ HSM mean	0.067	0.014	0.683	0.032	1341.226
p ~ HSM mean * biome	0.346	0.024	0.772	0.038	1116.217
p ~ HSM mean * functional type	0.189	0.017	0.683	0.036	1268.188
p ~ HSM min	0.264	0.022	0.750	0.033	1171.312
p ~ HSM min * biome	0.369	0.022	0.793	0.030	1091.891
p ~ HSM min * functional type	0.340	0.019	0.766	0.029	1125.860
p ~ sqrt(Number spp. HSM<0)	0.357	0.017	0.817	0.020	1865.168
p ~ sqrt(Number spp. HSM<0) * biome	0.434	0.020	0.826	0.025	1022.707
p ~ sqrt(Number spp. HSM<0) * functional type	0.470	0.014	0.836	0.019	1680.722
p ~ ln(HSM variance)	0.236	0.019	0.751	0.033	1197.921
p ~ ln(HSM variance) * biome	0.343	0.022	0.779	0.033	1118.953
p ~ ln(HSM variance) * functional type	0.460	0.010	0.828	0.021	1699.935

Supplementary Table 3. Relationship of hydraulic risk and mortality including aridity index as a covariable.

Variables significance (mean of the ANOVA test results for 100 models with different background points in each case) for logistic models with mortality occurrence as a response variable (binary) including a climate index as predictor (aridity index). I n the column 'variable', interaction is shown by ":". Significance codes: "***": $p < 0.0001$; "**": $p < 0.001$, "*": $p < 0.05$; NS: $p > 0.05$.

Model	Variable	df	Deviance mean	Resid. df mean	Resid. dev mean	Pr. Chi mean	Devianc e sd	Resid dev sd	Pr. Chi sd	Pr. Chi mean significance
p ~ HSM mean + ln(aridity index)	HSM mean	1	53.534	1000.000	1335.533	0.000	10.598	10.598	0.000	***
p ~ HSM mean + ln(aridity index)	ln(aridity index)	1	3.053	999.000	1332.480	0.223	2.994	10.306	0.214	NS
p ~ HSM mean * biome + ln(aridity index)	biome	6	211.323	994.000	1125.997	0.000	18.644	18.498	0.000	***
p ~ HSM mean * biome + ln(aridity index)	HSM mean	1	51.746	1000.000	1337.321	0.000	9.426	9.426	0.000	***
p ~ HSM mean * biome + ln(aridity index)	HSM mean:biome	6	21.390	987.000	1103.398	0.005	4.441	18.717	0.006	*
p ~ HSM mean * biome + ln(aridity index)	ln(aridity index)	1	1.210	993.000	1124.787	0.434	1.390	18.695	0.283	NS
p ~ HSM mean * functional type + ln(aridity index)	functional type	7	32.952	993.000	1303.902	0.001	7.681	11.205	0.002	**
p ~ HSM mean * functional type + ln(aridity index)	HSM mean	1	52.213	1000.000	1336.854	0.000	9.682	9.682	0.000	***
p ~ HSM mean * functional type + ln(aridity index)	HSM mean:functional type	7	61.136	985.000	1232.568	0.000	11.531	15.708	0.000	***
p ~ HSM mean * functional type + ln(aridity index)	ln(aridity index)	1	10.198	992.000	1293.704	0.014	5.132	10.846	0.036	.
p ~ ln(HSM variance) + ln(aridity index)	ln(aridity index)	1	7.026	999.000	1188.284	0.038	3.566	15.967	0.078	.
p ~ ln(HSM variance) + ln(aridity index)	ln(HSM variance)	1	193.757	1000.000	1195.310	0.000	16.515	16.515	0.000	***
p ~ ln(HSM variance) * biome + ln(aridity index)	biome	6	81.768	994.000	1110.967	0.000	12.346	20.381	0.000	***
p ~ ln(HSM variance) * biome + ln(aridity index)	ln(aridity index)	1	1.393	993.000	1109.574	0.425	1.661	20.705	0.298	NS
p ~ ln(HSM variance) * biome + ln(aridity index)	ln(HSM variance)	1	196.332	1000.000	1192.735	0.000	16.900	16.900	0.000	***
p ~ ln(HSM variance) * biome + ln(aridity index)	ln(HSM variance):biome	6	21.237	987.000	1088.337	0.009	5.625	23.029	0.017	*
p ~ ln(HSM variance) * functional type + ln(aridity index)	functional type	7	112.556	1723.000	1823.255	0.000	15.843	24.798	0.000	***
p ~ ln(HSM variance) * functional type + ln(aridity index)	ln(aridity index)	1	0.883	1722.000	1822.372	0.534	1.235	24.904	0.296	NS
p ~ ln(HSM variance) * functional type + ln(aridity index)	ln(HSM variance)	1	465.251	1730.000	1935.811	0.000	26.478	26.478	0.000	***

p ~ ln(HSM variance) * functional type + ln(aridity index)	ln(HSM variance):functional type	7	152.823	1715.000	1669.549	0.000	15.838	22.053	0.000	***
p ~ HSM min + ln(aridity index)	HSM min	1	220.492	1000.000	1168.575	0.000	19.283	19.283	0.000	***
p ~ HSM min + ln(aridity index)	ln(aridity index)	1	0.646	999.000	1167.930	0.564	0.830	19.157	0.271	NS
p ~ HSM min * biome + ln(aridity index)	biome	6	92.926	994.000	1074.115	0.000	14.567	21.186	0.000	***
p ~ HSM min * biome + ln(aridity index)	HSM min	1	222.026	1000.000	1167.041	0.000	19.720	19.720	0.000	***
p ~ HSM min * biome + ln(aridity index)	HSM min:biome	6	10.618	987.000	1062.268	0.168	3.806	22.782	0.164	NS
p ~ HSM min * biome + ln(aridity index)	ln(aridity index)	1	1.228	993.000	1072.887	0.460	1.554	21.716	0.301	NS
p ~ HSM min * functional type + ln(aridity index)	functional type	7	16.154	993.000	1151.774	0.069	5.272	18.874	0.092	NS
p ~ HSM min * functional type + ln(aridity index)	HSM min	1	221.139	1000.000	1167.928	0.000	19.708	19.708	0.000	***
p ~ HSM min * functional type + ln(aridity index)	HSM min:functional type	7	57.426	985.000	1093.693	0.000	10.881	18.582	0.000	***
p ~ HSM min * functional type + ln(aridity index)	ln(aridity index)	1	0.655	992.000	1151.119	0.540	0.761	19.016	0.256	NS
p ~ sqrt(Number spp. HSM<0) + ln(aridity index)	ln(aridity index)	1	41.829	1729.000	1824.266	0.000	12.322	30.667	0.000	***
p ~ sqrt(Number spp. HSM<0) + ln(aridity index)	sqrt(Number spp. HSM<0)	1	534.967	1730.000	1866.095	0.000	29.005	29.005	0.000	***
p ~ sqrt(Number spp. HSM<0) * biome + ln(aridity index)	biome	6	208.016	1724.000	1654.678	0.000	21.649	27.566	0.000	***
p ~ sqrt(Number spp. HSM<0) * biome + ln(aridity index)	ln(aridity index)	1	10.868	1723.000	1643.811	0.014	5.655	29.018	0.038	.
p ~ sqrt(Number spp. HSM<0) * biome + ln(aridity index)	sqrt(Number spp. HSM<0)	1	538.368	1730.000	1862.694	0.000	24.858	24.858	0.000	***
p ~ sqrt(Number spp. HSM<0) * biome + ln(aridity index)	sqrt(Number spp. HSM<0):biome	6	54.857	1717.000	1588.953	0.000	11.422	29.192	0.000	***
p ~ sqrt(Number spp. HSM<0) * functional type + ln(aridity index)	functional type	7	46.839	1723.000	1813.096	0.000	8.278	28.769	0.000	***
p ~ sqrt(Number spp. HSM<0) * functional type + ln(aridity index)	ln(aridity index)	1	39.652	1722.000	1773.444	0.000	10.485	27.221	0.000	***
p ~ sqrt(Number spp. HSM<0) * functional type + ln(aridity index)	sqrt(Number spp. HSM<0)	1	541.126	1730.000	1859.935	0.000	30.292	30.292	0.000	***
p ~ sqrt(Number spp. HSM<0) * functional type + ln(aridity index)	sqrt(Number spp. HSM<0):functional type	7	138.170	1715.000	1635.274	0.000	18.109	27.114	0.000	***

Supplementary Table 4. Number of mortality events observed per biome.

Biome reclassification and number of mortality observations per biome.

Aggregated biome	Biomes included	Number of total mortality points (1 per km^2)	Number of aggregated mortality points (1 per 10km^2)
Boreal	Tundra, Boreal Forests/Taiga,	39	34
Desert and xeric	Deserts & Xeric Shrublands	44	20
Mediterranean	Mediterranean Forests, Woodlands & Scrub,	292	139
Others	Montane Grasslands & Shrublands, Flooded Grasslands & Savannas, Mangroves,	4	4
Temperate	Temperate Grasslands, Savannas & Shrublands, Temperate Conifer Forests, Temperate Broadleaf & Mixed Forests	357	223
Tropical and subtropical dry	Tropical & Subtropical Grasslands, Savannas & Shrublands, Tropical & Subtropical Dry Broadleaf Forests, Tropical & Subtropical Coniferous Forests	73	42
Tropical and subtropical moist	Tropical & Subtropical Moist Broadleaf Forests,	73	55

Supplementary Table 5. Trends in the relationship between species assemblages hydraulic metrics and DIM occurrence as reported by applying the emmeans R package[2] to generalized linear models results.

Model	Factor	Trend mean	St error mean	P value mean	Trend sd	St error sd	P value sd	Mean p value significance
p ~ HSM mean * biome	Boreal	-0.345	0.772	0.616	0.391	0.057	0.250	NS
p ~ HSM mean * biome	Desert and xeric	-0.029	0.312	0.695	0.157	0.026	0.200	NS
p ~ HSM mean * biome	Mediterranean	-1.395	0.689	0.174	1.089	0.126	0.263	NS
p ~ HSM mean * biome	Others	1.412	1.483	0.421	1.170	0.492	0.203	NS
p ~ HSM mean * biome	Temperate	-2.473	0.338	0.000	0.307	0.014	0.000	***
p ~ HSM mean * biome	Tropical and subtropical dry	-1.886	0.922	0.068	0.531	0.066	0.084	NS
p ~ HSM mean * biome	Tropical and subtropical moist	-0.869	0.812	0.349	0.534	0.053	0.268	NS
p ~ HSM mean * functional type	Broadleaved deciduous	-2.257	0.823	0.014	0.595	0.091	0.021	.
p ~ HSM mean * functional type	Broadleaved evergreen	-6.622	0.936	0.000	0.866	0.090	0.000	***
p ~ HSM mean * functional type	Crops and Grassland	-1.489	0.334	0.000	0.275	0.021	0.001	**
p ~ HSM mean * functional type	Mixed forest	-1.321	1.363	0.383	1.014	0.188	0.255	NS
p ~ HSM mean * functional type	Mosaic	0.686	0.388	0.095	0.163	0.030	0.068	NS

p ~ HSM mean * functional type	Needleleaved	-8.505	0.953	0.000	0.936	0.106	0.000	***
p ~ HSM mean * functional type	Others	-1.338	0.616	0.050	0.423	0.082	0.047	.
p ~ HSM mean * functional type	Shrubland	-1.117	0.516	0.075	0.418	0.054	0.102	NS
p ~ HSM min * biome	Boreal	-0.416	0.288	0.202	0.168	0.023	0.153	NS
p ~ HSM min * biome	Desert and xeric	-0.493	0.134	0.003	0.152	0.017	0.009	*
p ~ HSM min * biome	Mediterranean	-1.043	0.282	0.022	0.351	0.041	0.082	.
p ~ HSM min * biome	Others	0.209	0.576	0.688	0.203	0.158	0.161	NS
p ~ HSM min * biome	Temperate	-0.705	0.076	0.000	0.048	0.003	0.000	***
p ~ HSM min * biome	Tropical and subtropical dry	0.085	0.073	0.294	0.035	0.002	0.202	NS
p ~ HSM min * biome	Tropical and subtropical moist	-0.323	0.070	0.000	0.029	0.001	0.000	***
p ~ HSM min * functional type	Broadleaved deciduous	-0.422	0.138	0.008	0.130	0.016	0.011	*
p ~ HSM min * functional type	Broadleaved evergreen	-0.248	0.068	0.016	0.071	0.003	0.070	.
p ~ HSM min * functional type	Crops and Grassland	-0.139	0.064	0.070	0.046	0.002	0.099	NS
p ~ HSM min * functional type	Mixed forest	-1.279	0.431	0.007	0.405	0.101	0.010	*
p ~ HSM min * functional type	Mosaic	-0.120	0.094	0.285	0.064	0.005	0.237	NS
p ~ HSM min * functional type	Needleleaved	-2.108	0.236	0.000	0.232	0.035	0.000	***
p ~ HSM min * functional type	Others	-0.442	0.118	0.001	0.098	0.011	0.002	**
p ~ HSM min * functional type	Shrubland	0.004	0.073	0.574	0.059	0.003	0.262	NS
p ~ sqrt(N spp. HSM < 0) * biome	Boreal	1.058	0.321	0.003	0.192	0.016	0.004	*
p ~ sqrt(N spp. HSM < 0) * biome	Desert and xeric	0.656	0.170	0.000	0.095	0.011	0.001	**
p ~ sqrt(N spp. HSM < 0) * biome	Mediterranean	1.526	0.296	0.000	0.270	0.035	0.000	***
p ~ sqrt(N spp. HSM < 0) * biome	Others	0.133	2.281	0.790	0.804	16.627	0.182	NS
p ~ sqrt(N spp. HSM < 0) * biome	Temperate	0.832	0.080	0.000	0.039	0.002	0.000	***
p ~ sqrt(N spp. HSM < 0) * biome	Tropical and subtropical dry	0.251	0.098	0.023	0.051	0.002	0.045	.
p ~ sqrt(N spp. HSM < 0) * biome	Tropical and subtropical moist	1.449	0.280	0.000	0.141	0.023	0.000	***
p ~ sqrt(N spp. HSM < 0) * functional type	Broadleaved deciduous	0.755	0.157	0.000	0.090	0.009	0.000	***
p ~ sqrt(N spp. HSM < 0) * functional type	Broadleaved evergreen	2.381	0.324	0.000	0.196	0.021	0.000	***
p ~ sqrt(N spp. HSM < 0) * functional type	Crops and Grassland	0.397	0.078	0.000	0.046	0.002	0.000	***
p ~ sqrt(N spp. HSM < 0) * functional type	Mixed forest	1.367	0.415	0.002	0.342	0.088	0.002	*
p ~ sqrt(N spp. HSM < 0) * functional type	Mosaic	0.346	0.138	0.028	0.075	0.005	0.052	.
p ~ sqrt(N spp. HSM < 0) * functional type	Needleleaved	2.280	0.264	0.000	0.137	0.018	0.000	***
p ~ sqrt(N spp. HSM < 0) * functional type	Others	0.641	0.155	0.000	0.087	0.007	0.000	**
p ~ sqrt(N spp. HSM < 0) * functional type	Shrubland	0.332	0.109	0.011	0.082	0.004	0.023	.
p ~ ln(HSM variance) * biome	Boreal	0.649	0.248	0.014	0.104	0.013	0.013	.
p ~ ln(HSM variance) * biome	Desert and xeric	1.032	0.414	0.031	0.302	0.046	0.053	.
p ~ ln(HSM variance) * biome	Mediterranean	2.817	0.561	0.000	0.457	0.070	0.000	***
p ~ ln(HSM variance) * biome	Others	2.198	1.300	0.104	0.612	0.210	0.050	NS
p ~ ln(HSM variance) * biome	Temperate	1.406	0.159	0.000	0.080	0.004	0.000	***
p ~ ln(HSM variance) * biome	Tropical and subtropical dry	0.008	0.130	0.704	0.061	0.007	0.183	NS
p ~ ln(HSM variance) * biome	Tropical and subtropical moist	1.172	0.266	0.000	0.103	0.008	0.000	***
p ~ ln(HSM variance) * functional type	Broadleaved deciduous	1.181	0.290	0.000	0.153	0.019	0.000	**
p ~ ln(HSM variance) * functional type	Broadleaved evergreen	3.604	0.436	0.000	0.175	0.017	0.000	***
p ~ ln(HSM variance) * functional type	Crops and Grassland	0.552	0.129	0.000	0.069	0.004	0.000	**

p ~ ln(HSM variance) * functional type	Mixed forest	3.790	1.361	0.009	1.251	0.378	0.012	*
p ~ ln(HSM variance) * functional type	Mosaic	0.704	0.254	0.013	0.152	0.015	0.021	.
p ~ ln(HSM variance) * functional type	Needleleaved	3.036	0.359	0.000	0.182	0.016	0.000	***
p ~ ln(HSM variance) * functional type	Others	0.869	0.281	0.003	0.103	0.015	0.003	*
p ~ ln(HSM variance) * functional type	Shrubland	0.121	0.138	0.416	0.101	0.007	0.275	NS

References

1. Trugman, A. T., Anderegg, L. D. L., Shaw, J. D. & Anderegg, W. R. L. Trait velocities reveal that mortality has driven widespread coordinated shifts in forest hydraulic trait composition. *Proc. Natl. Acad. Sci.* 201917521 (2020) doi:10.1073/pnas.1917521117.

2. Lenth, R. V. emmeans: Estimated Marginal Means, aka Least-Squares Means. R package version 1.6.3. (2021).
