# Supplementary material for: Increased hydraulic risk in assemblages of woody plant species predicts spatial patterns of drought-induced mortality
Source: Nat Ecol Evol. 2023 Aug 28;7(10):1620–32. doi: 10.1038/s41559-023-02180-z (PMC10555820; doi:10.1038/s41559-023-02180-z)
Supplement: Supplementary file 2 — Reporting Summary [file 41559_2023_2180_MOESM2_ESM.pdf]

Corresponding author(s): Pablo Sanchez-Martinez

Last updated by author(s): Jul 6, 2023

Reporting Summary

Nature Portfolio wishes to improve the reproducibility of the work that we publish. This form provides structure for consistency and transparency in reporting. For further information on Nature Portfolio policies, see our Editorial Policies and the Editorial Policy Checklist.

Statistics

For all statistical analyses, confirm that the following items are present in the figure legend, table legend, main text, or Methods section.

n/a	Confirmed	
☐	☒	The exact sample size (*n*) for each experimental group/condition, given as a discrete number and unit of measurement
☐	☒	A statement on whether measurements were taken from distinct samples or whether the same sample was measured repeatedly
☐	☒	The statistical test(s) used AND whether they are one- or two-sided<br>*Only common tests should be described solely by name; describe more complex techniques in the Methods section.*
☐	☒	A description of all covariates tested
☐	☒	A description of any assumptions or corrections, such as tests of normality and adjustment for multiple comparisons
☐	☒	A full description of the statistical parameters including central tendency (e.g. means) or other basic estimates (e.g. regression coefficient) AND variation (e.g. standard deviation) or associated estimates of uncertainty (e.g. confidence intervals)
☐	☒	For null hypothesis testing, the test statistic (e.g. *F*, *t*, *r*) with confidence intervals, effect sizes, degrees of freedom and *P* value noted<br>*Give P values as exact values whenever suitable.*
☐	☒	For Bayesian analysis, information on the choice of priors and Markov chain Monte Carlo settings
☐	☒	For hierarchical and complex designs, identification of the appropriate level for tests and full reporting of outcomes
☐	☒	Estimates of effect sizes (e.g. Cohen's *d*, Pearson's *r*), indicating how they were calculated

Our web collection on statistics for biologists contains articles on many of the points above.

Software and code

Policy information about availability of computer code

Data collection	Data used come from global databases which have been compiled by authors in previous works. More details on data measurement can be found in the methods section of the present work and the previously published ones describing the data used. The minimum dataset needed to replicate analyses can be found in DOI: 10.6084/m9.figshare.23635446.
Data analysis	The code for data analyses can be found in the following repository: https://github.com/pablosanchezmart/Sanchez-Martinez_etal-2022.

For manuscripts utilizing custom algorithms or software that are central to the research but not yet described in published literature, software must be made available to editors and reviewers. We strongly encourage code deposition in a community repository (e.g. GitHub). See the Nature Portfolio guidelines for submitting code & software for further information.

Data

Policy information about availability of data

All manuscripts must include a data availability statement. This statement should provide the following information, where applicable:

- Accession codes, unique identifiers, or web links for publicly available datasets
- A description of any restrictions on data availability
- For clinical datasets or third party data, please ensure that the statement adheres to our policy

The code used can be found in https://github.com/pablosanchezmart/Sanchez-Martinez_etal-2022. All data needed to replicate the analyses is available in previous

publications except for the hydraulics dataset, which will be published soon by William Hammond and collaborators in a data paper. The minimum dataset needed to replicate analyses can be found in DOI: 10.6084/m9.figshare.23635446.

Human research participants

Policy information about studies involving human research participants and Sex and Gender in Research.

Reporting on sex and gender	*Use the terms sex (biological attribute) and gender (shaped by social and cultural circumstances) carefully in order to avoid confusing both terms. Indicate if findings apply to only one sex or gender; describe whether sex and gender were considered in study design whether sex and/or gender was determined based on self-reporting or assigned and methods used. Provide in the source data disaggregated sex and gender data where this information has been collected, and consent has been obtained for sharing of individual-level data; provide overall numbers in this Reporting Summary. Please state if this information has not been collected. Report sex- and gender-based analyses where performed, justify reasons for lack of sex- and gender-based analysis.*
Population characteristics	*Describe the covariate-relevant population characteristics of the human research participants (e.g. age, genotypic information, past and current diagnosis and treatment categories). If you filled out the behavioural & social sciences study design questions and have nothing to add here, write "See above."*
Recruitment	*Describe how participants were recruited. Outline any potential self-selection bias or other biases that may be present and how these are likely to impact results.*
Ethics oversight	*Identify the organization(s) that approved the study protocol.*

Note that full information on the approval of the study protocol must also be provided in the manuscript.

Field-specific reporting

Please select the one below that is the best fit for your research. If you are not sure, read the appropriate sections before making your selection.

☐ Life sciences ☐ Behavioural & social sciences ☒ Ecological, evolutionary & environmental sciences

For a reference copy of the document with all sections, see nature.com/documents/nr-reporting-summary-flat.pdf

Ecological, evolutionary & environmental sciences study design

All studies must disclose on these points even when the disclosure is negative.

Study description	Here, we use a newly global database to predict hydraulic risk of woody plant species using Random Forests. Then, we use generalized linear models to test whether hydraulic risk predict observed drought induced mortality (DIM). Finally, we project DIM occurrence probability worldwide using maximum entropy models.
Research sample	Hydraulics data comes from a global dataset on hydraulic traits which will be soon published by William Hammond. A previous version of this dataset can be found, for instance, in Sanchez-Martinez et al. 2020. Species distribution data comes from a compilation and generalization of global data on species occurrences (see Serra-Diaz et al. 2017). Mortality data comes from a global dataset compiling drought induced mortality occurrence (Hammond et al. 2022). All data sources are referenced in the main text.
Sampling strategy	In this global study, all available data that passed a quality filter (see methods) was used.
Data collection	Data collection information can be found in the cited papers which describe datasets used in this study.
Timing and spatial scale	Data used comes from peer reviewed publications reporting hydraulic traits, drought induced mortality and species distributions. Then, there is not a specific team scale. the spatial scale is global.
Data exclusions	For hydraulic traits, only data coming from stems of mature individuals were used. P50 and P88 included only observations with values < -0.5MPa that originated from S-shaped vulnerability curves, as these values are prone to present a high measurement error.
Reproducibility	No experiment was conducted in the present study, as it is a global analyses.
Randomization	No experiment was conducted in the present study, as it is a global analyses.
Blinding	No experiment was conducted in the present study, as it is a global analyses.

Did the study involve field work? ☐ Yes ☒ No

Reporting for specific materials, systems and methods

We require information from authors about some types of materials, experimental systems and methods used in many studies. Here, indicate whether each material, system or method listed is relevant to your study. If you are not sure if a list item applies to your research, read the appropriate section before selecting a response.

Materials & experimental systems

n/a	Involved in the study
☒	☐ Antibodies
☒	☐ Eukaryotic cell lines
☒	☐ Palaeontology and archaeology
☒	☐ Animals and other organisms
☒	☐ Clinical data
☒	☐ Dual use research of concern

Methods

n/a	Involved in the study
☒	☐ ChIP-seq
☒	☐ Flow cytometry
☒	☐ MRI-based neuroimaging
